# College and Hospital Notes

**Published:** 1904-03

**Authors:** 


### COLLEGE AND HOSPITAL NOTES.

The Children's Hospital, of Buffalo, has issued its eleventh annual report, which covers the period for the year ending October 1, 1903. This hospital is one of the most deserving charities and is doing a splendid work. The demands upon its space and purse are increasing with the growth of the city, and philanthropic people are invited to contribute to the support of the hospital.

---

The New York State Hospital for the care of Crippled and Deformed Children, located at Tarrytown, recently has sent out its third annual report, which is for the year ending September 30, 1903. The Right Rev. Henry C. Potter, D. D., Bishop of New York, is president of the board of managers and Dr. Newton M. Shaffer, of New York, is the surgeon-in-chief of the hospital. Buffalo University Medical College is represented on the consulting staff by Drs. Roswell Park and Charles G. Stockton. The board of managers express the wish that the names and addresses of poor children residing in Buffalo and vicinity, who are crippled or deformed, be sent to the surgeon-in-chief of the hospital at Tarrytown.

---

The Buffalo General Hospital, according to the report of Stephen M. Clement, Esq., president, lately published, has raised during the past year $185,242.50 for the purpose of liquidating certain indebtedness which has acumulated during several years past. This magnificent sum has been raised by subscription and reflects great credit upon the astute financial management of Mr. Clement. Two subscriptions are notable, that of Mr. F. H. Goodyear for $50,000 and that of Mr. William Hamlin for $49,000. The hospital still needs about $15,000 to make good the deficit. It seems probable that this comparatively small sum will be contributed by some of our philanthropic citizens.

---

At Hagerstown, Md., a hospital is to be established for the county of Washington. A public meeting was held February 3, 1904, at which it was voted to build a hospital to cost $50,000, with a maintenance fund for annual current expenses estimated at $10,000. Taxes will be levied to meet these items of expenditure. If this kind of enterprise prevailed in the state of New York, there would be less solicitation of private subscriptions to meet the cost of maintaining public charities, among which every general hospital should be classed.

---

At the German Deaconess's Hospital, 218 Kingsley street, Buffalo, a vacancy has occurred on the house staff. Young physicians who desire such an appointment may address in person or in writing the superintendent of the hospital as above indicated.

University day, February 22, 1904, was celebrated by the students in the several departments of the University of Buffalo in a fitting manner. Under the escort of a brass band they marched in a body from the university building to Concert Hall, where they listened to an address by George E. Vincent, P. H. D., of the University of Chicago, on the subject, Education and efficiency. The proceedings were opened with prayer by the Rev. Charles Edward Locke, after which Dr. M. D. Mann, dean of the medical department, introduced the speaker. The proceedings were interspersed by musical selections rendered by the University Mandolin Club. Afterward, Dr. Vincent was entertained at luncheon at the Saturn Club by the faculty of the University.

---

The Riverside Hospital has issued its annual announcement for 1904. It is located at 113 Lafayette avenue, Buffalo, a residential section of the city and is owned and managed by Dr. Lillian Craig Randall, who established it about twelve years ago. The hospital has accommodations for forty patients and is equipped with the usual operating, electrical, sterilising, and anesthetising rooms. It has an out-patient department, also an accident hospital, the latter being located at 118 East Swan street. It also has a first-relief station located at Black Rock. There is a maternity department attached to the out-patient service under the charge of Dr. James E. King. Applications for admission to the Riverside Hospital or any of its branches, should be made to Dr. Randall.

